# miRNA-660-3p inhibits malignancy in glioblastoma via negative regulation of APOC1-TGFβ2 signaling pathway

**DOI:** 10.1080/15384047.2023.2281459

**Published:** 2023-11-19

**Authors:** Zelin Yang, Liang Yang, Zhenkai Sun, Yuxi Rong, Chenglian Bai, Qiaoxiang Dong, Lin Jian

**Affiliations:** a Department of Neurosurgery, The Second Affiliated Hospital and Yuying Children’s Hospital, Wenzhou Medical University, Wenzhou, China; b School of Public Health and Management, Wenzhou Medical University, Wenzhou, China

**Keywords:** Glioblastoma, APOC1, miRNA-660-3p, TGFβ2 signaling pathway

## Abstract

Glioblastoma as the most common and aggressive central nervous system tumor in adults. Its prognosis and therapeutic outcome are poor due to the limited understanding of its molecular mechanism. Apolipoprotein C-1 (APOC1) as a member of the apolipoprotein family that acts as a tumor promoter in various cancers. MicroRNA (miRNA) can silence gene expression and suppress tumor progression. However, the role of APOC1 and its upstream miRNA has not been explored in glioblastoma. Two glioblastoma cell lines (U87 and U251) were used to explore the role of APOC1 and its upstream miRNA-660-3p in glioblastoma tumorigenesis *in vitro*. Cells with APOC1/miRNA-660-3p overexpression or knockdown were assessed for their proliferation, migration, and invasion *in vitro*, and tumorigenesis *in vivo*. Gene and protein expression was assessed by qRT-PCR and western blot, respectively. Cell proliferation was assessed by the MTT assay and the EdU and Ki67 staining. Cell migration and invasion were assessed by the transwell assay. Tumorigenesis *in vivo* was assessed in U87 cells with a xenograft mouse model. APOC1 was overexpressed in glioblastoma compared with normal peritumoral tissue and was inversely related to patient prognosis. APOC1 overexpression promotes cell proliferation, migration, and invasion *in vitro*. APOC1 inhibition reduced tumor growth in *vivo*. miRNA-660-3p inhibits tumorigenesis by directly targeting APOC1. Mechanistically, APOC1 drives the malignancy of glioblastoma by activating the TGFβ2 signaling pathway. miRNA-660-3p suppresses tumorigenesis by targeting APOC1. Therefore, miRNA-660-3p/APOC1 axis can serve as potential intervention targets in managing glioblastoma progression.

## Introduction

Glioblastoma is one of the most aggressive intracranial tumors in the adult central nervous system (CNS), accounting for approximately 14.3% of all primary CNS tumors.^1^ Glioblastoma is characterized by its high invasion and vascularization^2–4^ and the rapid progression that ultimately leads to poor prognosis.^5^ The median survival time for most patients under the standard care of surgical resection combined with adjuvant radiotherapy is approximately 12 months.^2,6^ However, even with the most recent advance of combined therapy involving maximal surgical resection followed by radiotherapy and temozolomide, almost all patients experience tumor progression with a median survival time of fewer than 15 months.^7,8^ In addition, drug resistance often occurs in glioblastoma patients due to overexpressed anti-apoptotic proteins.^5^ Generally, the expected survival time for glioblastoma patients is about two years after diagnosis.^9^ The poor prognosis and high mortality are mainly due to a limited understanding of disease progression in glioblastoma. Therefore, it is vital to study the molecular mechanisms underlying tumor progression in glioblastoma, which will help us develop better therapeutic interventions for disease control in these patients.

Apolipoprotein C-1 (APOC1) is a member of the apolipoprotein family that stabilize lipoprotein structure in lipid transport but also participate in pathological processes leading to many diseases such as diabetes, Alzheimer’s, and cancers.^10–13^ Apolipoproteins are proteins that bind lipids to form lipoproteins, functioning as lipid carriers. Recently, many apolipoproteins have been reported to be involved in tumor pathogenesis.^14^ For APOC1, early studies have shown that it can promote tumor progression in many malignancies, including gastric cancer,^15^ colorectal cancer,^16,17^ prostate cancer,^18^ breast cancer,^19^ cervical cancer,^20^ lung cancer,^21^ pancreatic cancer,^22^ and renal carcinoma.^23^ However, its role in tumor progression has not been explored in brain tumors, including glioblastoma.

MicroRNA (miRNA) is a cellular endogenous non-coding RNA molecule about 19–24 nucleotides in length^24^ regulating gene expression through binding to mRNA. miRNAs silence gene expression by targeting the complementary 3‘UTR site of the mRNA.^25^ Numerous research has indicated that the same miRNA can target and regulate multiple mRNAs in various biological processes such as apoptosis, DNA damage repair, proliferation, cell cycle, senescence, invasion, and angiogenesis.^26^ For example, in glioblastoma, miRNA-1 inhibited cell proliferation and migration *in vitro* and tumorigenesis *in vivo* by directly targeting fibronectin.^27^ In addition, cytosine-methylation of miRNA-181a-5p was associated with poor prognosis in glioblastoma patients.^28^ These findings indicate that miRNAs and their downstream genes can serve as potential drug targets for managing tumor progression. However, little research indicated the relationship between miR-660-3p and tumors. Only know is that miR-660-3p can be sponged by circRNA and then regulated drug resistance and malignancy in pancreatic cancer and breast cancer. However, there has not been any study exploring the potential relationship between miRNAs and APOC1 in glioblastoma tumorigenicity.

In this study, two glioblastoma cell lines (U87 and U251) were used to explore the role of APOC1 and miRNAs in glioblastoma tumorigenicity. First, the TCGA database was used to analyze the expression level of APOC1 and its association with prognosis in glioblastoma patients. Second, cells with APOC1 overexpression or knockdown were assessed for their proliferation, migration, and invasion *in vitro*, and tumorigenesis *in vivo* using a subcutaneous xenograft mouse model. Third, miRNA targeting APOC1 was identified, and its role in glioblastoma malignancy *in vitro* was assessed by transfecting cells with its mimic or inhibitor. Lastly, the downstream signaling pathway of APOC1 was uncovered and validated. Our study reveals that miRNA-660-3p inhibits the glioblastoma tumorigenesis by targeting the APOC1-TGFβ2 signaling pathway.

## Results

### APOC1 upregulation is negatively correlated with glioblastoma patient prognosis

**Figure 1.**
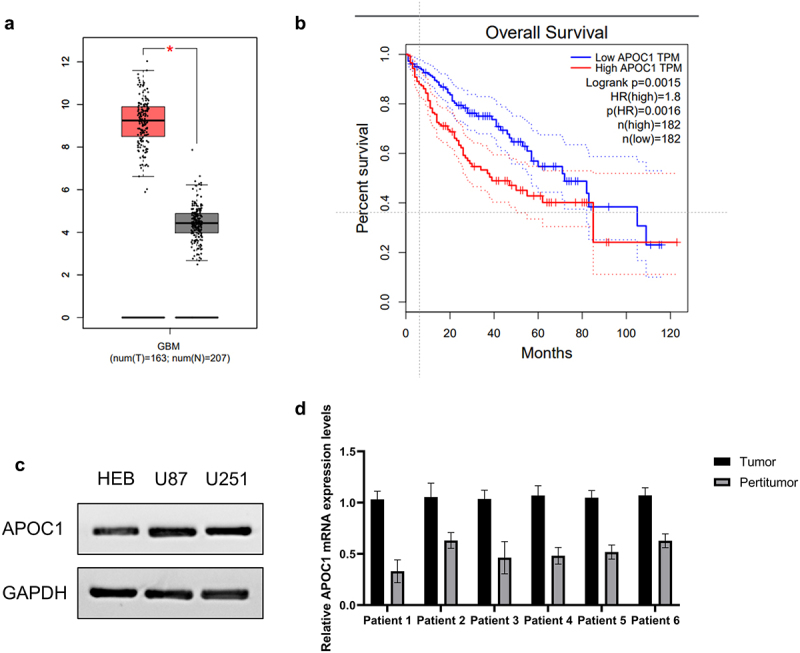
APOC1 expression in tumor and paraneoplastic tissues and its relationship with prognosis in glioblastoma patients.

To investigate the pathogenesis of glioblastoma, we screened the GEPIA (http://gepia.cancer-pku.cn/detail.php?gene=&clicktag=boxplot) database for genes encoding the apolipoprotein family in paired glioblastoma and paraneoplastic tissues. The expression of APOC1 was much higher in glioblastoma than in normal paraneoplastic tissue (Figure 1a). Furthermore, the Kaplan-Meier curves from TCGA database revealed a lower survival rate in glioblastoma patients with high APOC1 expression (Figure 1b), suggesting that APOC1 may serve as a potential pro-oncogene in the development of glioblastoma. In order to clarify the expression of APOC1 in glioblastoma, we detected the APOC1 protein expression level in U87, U251 and HEB cells. As predicted, APOC1 protein is upregulated in glioblastoma (Figure 1c). Further, we found APOC1 was consistently overexpressed in tumors when compared with peritumor tissues in samples collected from GBM patients (*n* = 6, Figure 1d).

a:Expression of APOC1 in glioblastoma and paracancerous tissues; *P < 0.05; b: Kaplan-Meier analysis shows reduced postoperative survival of glioblastoma patients with higher APOC1 expression (data extracted from the TCGA database). c: Expression level of APOC1 was detected in HEB, U87 and U251 cells by western blot. D: Expression of APOC1 mRNA was detected in six GBM patients’ tumor and peritumor tissues by RT-qPCR.

## APOC1 upregulation exacerbates glioblastoma cell malignancy *in vitro*

**Figure 2.**
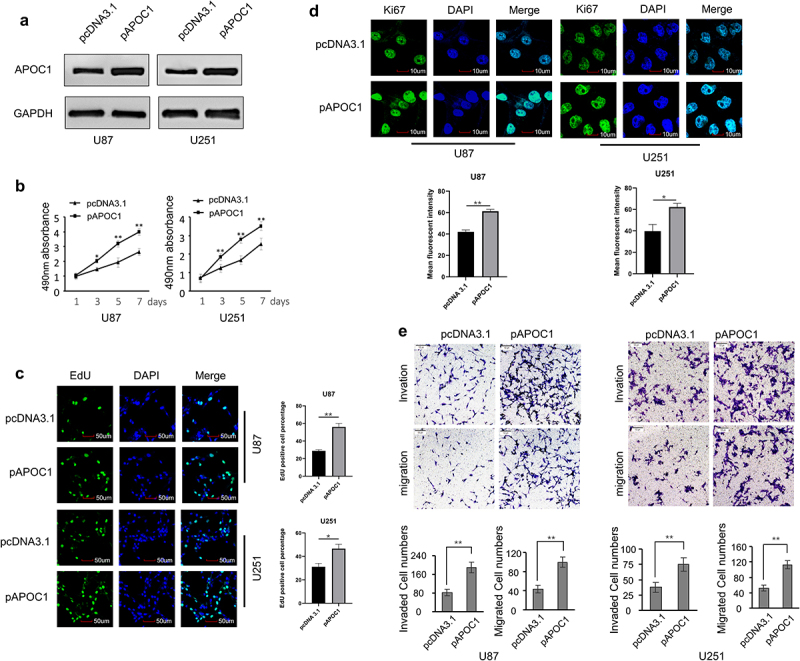
Overexpressed APOC1 promotes cell proliferation, migration, and invasion *in vitro.*

Glioblastoma cells (U87 and U251) transfected with APOC1 plasmid (pAPOC1) showed upregulated APOC1 mRNA and protein (Figure 2a, Figure S1A), increased cell proliferation (Figure 2b-d), and enhanced cell migration and invasion (Figure 2e). Cell proliferation was characterized by cell growth assessed by the MTT assay (Figure 2b) and DNA replication activity assessed by the EdU (Figure 2c) and Ki67 staining (Figure 2d). In addition, cell migration and invasion were detected by the transwell assay (Figure 2e). Conversely, cells transfected with APOC1 shRNA showed the exact opposite (Figure S1B, Figure S2).

a: Western blots show elevated APOC1 protein levels in U87 and U251 cells transfected with APOC1 plasmid (pAPOC1) when compared with those transfected with the control plasmid (pcDNA3.1); b-d: Cell proliferation assessed by the MTT assay (c), EdU immunofluorescent staining (c), and Ki67 immunofluorescent staining (d); e: Cell migration and invasion assessed by the transwell assay. *P < 0.05, and **P < 0.01 *vs*. PcDNA3.1 group.

### miRNA-660-3p directly targets the 3′-UTR of APOC1

**Figure 3.**
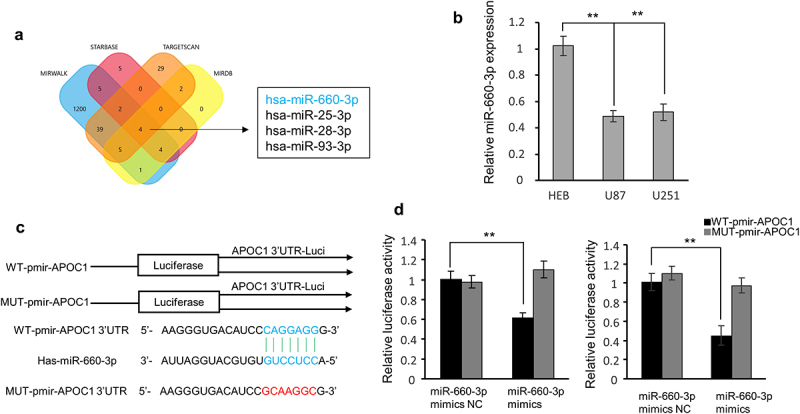
miRNA-660-3p directly targets the 3′-UTR of APOC1. a: Four-way Venn diagram shows predicted miRNAs that can bind to APOC1 based on four publicly available databases; b: miRNA-660-3p expression in normal HEB cells and glioblastoma cells (U87 and U251); c: Nucleotide prediction of APOC1 binding sites to miRNA-660-3p; d: The dual-luciferase reporter assay shows binding affinity of miRNA-660-3p with APOC1.

Binding site analysis using four bioinformatics databases (MIRWALK, Targetscan, DIANA, and RNA22) predicted miRNA-660-3p to be the potential miRNA that binds the 3’ -UTR of APOC1 (Figure 3a). RT-qPCR analysis showed decreased expression of miRNA-660-3p in U87 and U251 cells compared to normal HEB cells (Figure 3b). To further validate the predicted results, two reporter vectors with luciferase tagging sequences were constructed for the 3’-UTR of APOC1 (WT-pmir-APOC1 3‘UTR) and the mutant 3’-UTR (MUT-pmir-APOC1 3‘UTR) (Figure 3c). After transfection, miRNA-660-3p only reduced the luciferase activity of WT-pmir-APOC1 in both cell lines (Figure 3d), indicating that miRNA-660-3p directly binds to APOC1 mRNA.

### miRNA-660-3P inhibits glioblastoma malignancy *in vitro* by targeting APOC1

**Figure 4.**
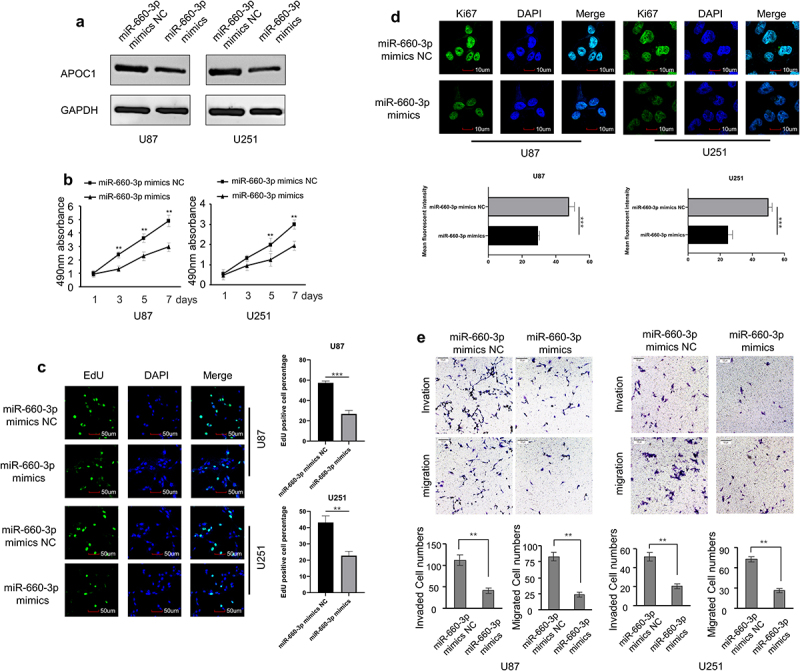
miRNA-660-3p inhibits cell proliferation, migration, and invasion *in vitro.*

Glioblastoma cells (U87 and U251) transfected with miR-660-3p mimic showed a significant reduction of APOC1 mRNA and protein (Figure 4a, Figure S1C). Consequently, cell proliferation, migration, and invasion were inhibited in miR-660-3p-overexpressed U87 and U251 cells (Figure 4b-e). Conversely, U87 and U251 cells transfected with miR-660-3p inhibitor showed opposite findings (Figure S3A-E, Figure S1D). Subsequently, APOC1 overexpression plasmid and miR-660-3p mimic were applied to co-transfect U87 and U251 cells for rescue experiments. The result showed upregulated APOC1 could promote the cell proliferation, migration and invasion ability, while the miR-660-3p could counteract the increased malignancy caused by overexpression of APOC1 (Figure S4A-C).

a: Western blots show decreased APOC1 protein levels in U87 and U251 cells transfected with miR-660-3p mimic when compared with those transfected with miR-660-3p mimic NC. (negative control); B-D: Cell proliferation assessed by the MTT assay (b), EdU immunofluorescent staining (c), and Ki67 immunofluorescent staining (d); e: Cell migration and invasion assessed by the transwell assay. **P < 0.01, and ***P < 0.001.

### miRNA-660-3P -APOC1 axis regulates glioblastoma tumorigenesis *in vivo*

**Figure 5.**
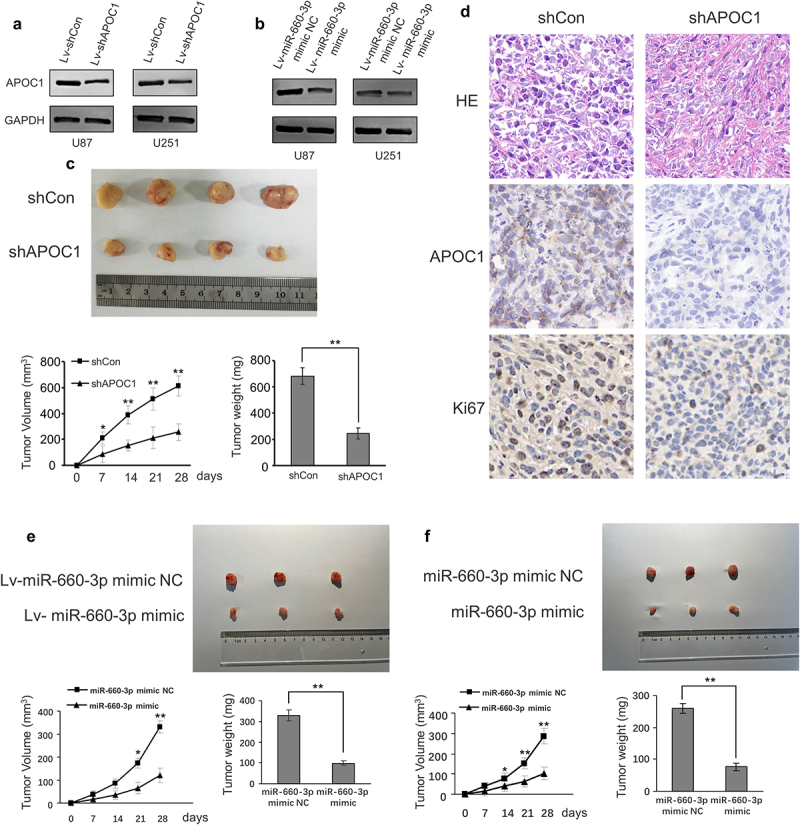
APOC1 knockdown decreases U87 cell tumorigenic ability *in vivo.*

Glioblastoma U87 and U251 cells with stably APOC1 knocked down (lv-shAPOC1) were constructed by transfecting lentivirus. The efficiency of APOC1 expression was detected by western blot (Figure 5a). Then, the lv-shAPOC1 U87 cell was injected subcutaneously into nude mice to assess tumorigenesis *in vivo*. Tumors in nude mice inoculated with lv-shAPOC1 U87 cells were smaller and had significantly lower volume and weight than those inoculated with the control plasmid (Figure 5c). In addition, immunohistochemical staining showed decreased expression of APOC1 and Ki67 in tissue sections of tumors originating from mice injected with lv-shAPOC1 U87 cells (Figure 5d). To determine whether miR-340-APOC1 axis affects tumor formation in vivo, we further performed the *in vivo* tumorigenesis study by subcutaneously injecting Lv-miR-660-3p-U87 and Lv-miR-660-3p-U87 cells into nude mice. The inhibitory effect of miR-660-3p on APOC1 expression was detected by Western blot (Figure 5b). The mice in the Lv-miR-660-3p and control groups were euthanized 28 days after inoculation, and the average tumor volumes and weights were significantly reduced in Lv-miR-660-3p groups (Figure 5(e,)).

**A**:The expression of APOC1 was detected by western blot. B: The expression of APOC1 after transfected by lv-miR-660-3p mimic NC and lv-miR-660-3p mimic were detected by western blot. C: Tumors excised from nude mice inoculated with U87 cells stably transfected with control (shCon) or APOC1 (shAPOC1) shRNA (*n* = 6, 4 of 6 were showed), with measurements of tumor volume and weight (*n* = 6). E: HE staining and immunohistochemistry of APOC1 and Ki67 in tumor tissue sections. E and F: Tumors excised from nude mice inoculated with U87 and U251 cells stably transfected with control (lv-miR-660-3p mimic NC) or miR-660-3p mimic (lv-miR-660-3p mimic) (*n* = 5, 3 of 5 were showed), and the measurements of tumor volume and weight (*n* = 5). *P < 0.05, and **P < 0.01.

### miRNA-660-3P-APOC1 axis negatively regulates the TGFβ2 signaling pathway

**Figure 6.**
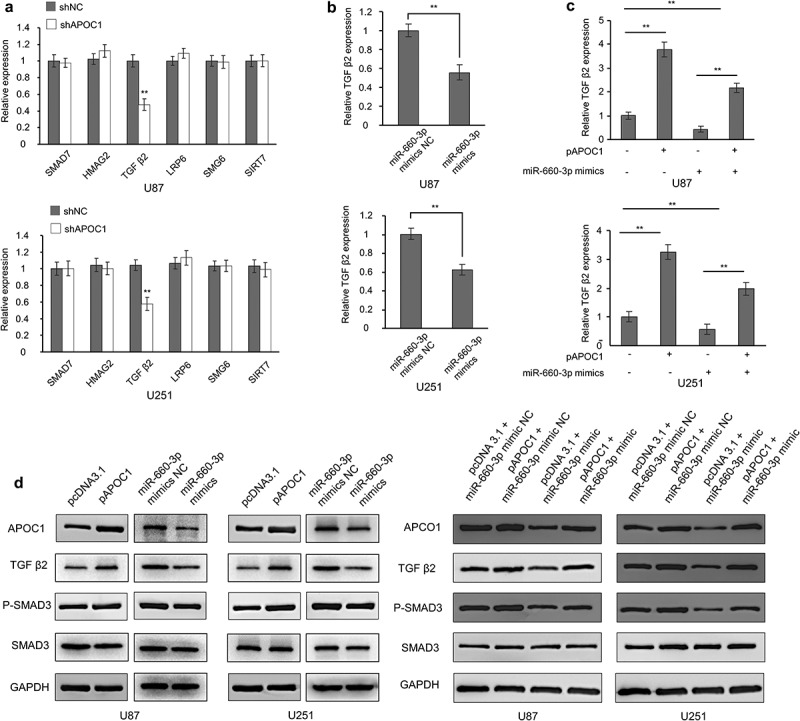
miRNA-660-3p-APOC1 axis negatively regulates the TGFβ2 pathway. a: Expression of essential genes of common tumor signaling pathways in U87 and U251 cells transfected with control (shCon) and APOC1 (shAPOC1) shRNA; B-C: Expression of TGFβ2 in U87 and U251 cells transfected with miR-660-3p mimic (b) or co-transfected with pAPOC1 and miR-660-3p mimic (c); d: Western blot to detect the expression of TGFβ2-associated protein after transfection with pAPOC1 and miR-660-3p mimic; (e) The effects of the miR-660-3p mimic and pAPOC1 on the protein levels of TGFβ2-related genes were evaluated by Western blot. **P < 0.01.

To explore the potential molecular mechanism by which APOC1 promotes glioblastoma malignancy, we examined some common tumor signaling pathways after the APOC1 was knocked down. Our analysis revealed significant downregulation of TGFβ2 in U87 and U251 cells (Figure 6a). Similarly, cells transfected with miR-660-3p mimic had decreased expression of TGFβ2 (Figure 6b). Meanwhile, the upregulated TGFβ2 caused by APOC1 overexpression was partially dampened after miR-660-3p mimic transfection (Figure 6c). Western blotting showed that both TGFβ2 and its downstream phospho-SMAD3 protein levels were elevated after overexpression of APOC1 and decreased after transfection of miR-660-3p mimic (Figure 6d). These results suggest that the miRNA-660-3P-APOC1 axis negatively regulates the TGFβ2 signaling pathway in glioblastoma cells. The rescue experiments showed the overexpressed APOC1 upregulated the protein expressions of TGFβ2 and p-SMAD3. Meanwhile, miR-660-3p can partially nullified the oncogenic effect of the APOC1 on glioblastoma cells.

## Discussion

This study showed that APOC1 is significantly elevated in human glioblastoma tissues, and its inhibition led to decreased cell proliferation, migration, and invasion *in vitro* and tumor growth *in vivo*, suggesting that APOC1 may serve as a driver gene in glioblastoma malignancy. In addition, miR-660-3p was found to target APOC1 mRNA and inhibit its expression, which in turn inhibits cell proliferation, migration, and invasion *in vitro*. Further, we discovered that APOC1 inhibition downregulates TGFβ2 and disrupts its associated downstream SMAD3 and pSMAD3, revealing a new signaling pathway involving the miR-660-3p/APOC1/TGFβ2 axis in the development of glioblastoma. Our findings suggest that miR-660-3p/APOC1/TGFβ2 axis may serve as a new potential therapeutic target for glioblastoma.

Previous studies had suggested that abnormalities in cholesterol biosynthesis and its downstream signaling can lead to glioblastoma tumorigenesis.^29^ Cholesterol metabolism in the zbrain is different from that in other organs because cholesterol in plasma cannot cross the blood-brain barrier. Instead, cholesterol in the brain is synthesized in astrocytes and delivered to neurons through the apolipoprotein E (APOE).^30^ Notably, a recent study showed that the demand for cholesterol in glioblastoma cells was mainly exogenous rather than endogenous,^31^ which has also been suspected to be the underlying cause of developing drug resistance in glioblastoma.^31^ This study also highlights the importance of cholesterol sources in glioblastoma progression. Together, these findings call for further exploration of cholesterol utilization in glioblastoma cells to unravel possible molecular mechanisms of tumor progression.

APOC1, as a member of the apolipoprotein family, is the primary apolipoprotein of the very-low-density lipoprotein (VLDL)-cholesterol complex.^32^ This may explain why APOC1 was commonly overexpressed in glioblastoma, as exogenous cholesterol is required for tumor growth. In addition, APOC1 has been shown to serve as a tumor-promoting factor in other malignancies. For example, in triple-negative breast cancer, APOC1 induced epithelial-mesenchymal transition (EMT) by suppressing E-calreticulin.^19^ In renal clear cell carcinoma, APOC1 directly enhanced the STAT3 signaling, which in turn increased cell migration and invasion through EMT.^23^ In pancreatic cancer, APOC1 suppressing through the BET inhibitor(JQ1s) enhanced the therapeutic effect of gemcitabine.^33^ More recently, APOC1 has been identified as a critical marker related to oncogenesis and prognosis of colorectal, cervical, and renal clear cell carcinoma.^17,20^ In the present study, we, for the first time, demonstrated that APOC1 promotes cell proliferation, migration, invasion *in vitro*, and tumor growth *in vivo* in glioblastoma. We only determined the tumor growth in vivo by the subcutaneous xenograft model in this research, However, evaluate the growth of glioblastoma by orthotopic xenograft model would be a better idea.

The non-coding miRNAs, similar to the transcription factors, play essential roles in gene expression regulation.^34^ Many studies have shown that miRNAs expression is closely related to tumorigenesis and malignancy.^35^ For example, in glioblastoma, miR-301a-3p promotes cell proliferation and invasion by upregulating FOSL1 expression through sponging with long non-coding RNA LncRNA-HOTAIR,^36^ and miR-3189 targets GLUT3 to inhibit cell proliferation by regulating glucose metabolism.^37^ In addition, APOC1 has been reported to serve as a direct target for miR-17 and miR-515 in breast and prostate cancer, respectively, to halt tumor growth.^38^ More recently, miR-660-3p was reported to be sponged by circFARP1 to upregulate LIF expression and inhibit gemcitabine resistance in pancreatic ductal adenocarcinoma.^39^ In breast cancer, circWWC3 upregulates multiple oncogenes’ expression of the Ras signaling pathway through acting as the sponge of miR-660-3p.^40^ In the present study, we demonstrated that miR-660-3p directly targeted APOC1 and inhibited APOC1 expression, subsequently leading to reduced cell proliferation, migration, and invasion in glioblastoma cells. The fact that miRNAs can cross the blood-brain barrier via exosome and transport between tumor cells^41^ makes miRNAs promising therapeutic targets for glioblastoma.

The transforming growth factor β (TGFβ) signaling pathway plays an indispensable role in the regulation of tumor malignancy, such as in breast cancer,^42^ non-small cell lung cancer,^43^ colorectal cancer,^44^ and many other tumors.^45–47^ Earlier studies have revealed that the TGFβ2 signaling pathway can be regulated by PDLIM5 to promote lung cancer migration and invasion.^43^ Furthermore, the expression TGFβ2 receptor and its signaling in glioblastoma are closely related to the patients’ prognosis.^48^ In addition, TGBβ2 downstream protein SMAD was also associated with glioblastoma malignancy.^49^ In this study, we revealed that TGFβ2 signaling is a downstream pathway for APOC1 in glioblastoma, and its suppression can reduce tumor progression.

This study revealed the role of APOC1 in promoting glioblastoma malignancy and the potential underlying molecular mechanism. APOC1 expression is negatively correlated to glioblastoma prognosis. APOC1 suppression through shRNA or miRNA-660-3p could lead to reduced cell proliferation, migration, and invasion in glioblastoma cells and reduced tumor growth in tumor xenografts. APOC1 suppression is also associated with the downregulation of the TGFβ2 signaling pathway. In conclusion, the miRNA-660-3p-APOC1 axis could inhibit the glioblastoma malignancy by negatively regulating the TGFβ2 signaling pathway. Targeting APOC1 or miRNA-660-3p may provide a new therapeutic intervention for glioblastoma.

## Materials and methods

### Cell lines and cell culture

Human glioblastoma cell lines (U87 and U251) and HEB cell line were purchased from the ATCC. U87, U251 and HEB cells has been performed authenticated using Short Tandem Repeat (STR) analysis on 2021 in Wuhan Procell Life Science&Technology Co., Ltd. The complete medium for U87, U251, and HEB consists of 90% DMEM medium (Gibco, NY, USA), 10% fetal bovine serum (FBS, Gibco, NY, USA) and 1% penicillin/streptomycin (Gibco, NY, USA). All cells were cultured at 37°C in a 5% CO_2_ incubator.

### Cell transfection

The control and APOC1 (Hanheng, Wuhan, China) downregulated stable cell lines were established using lentivirus transduction and labeled as sh-control and sh-APOC1, respectively. These cells were confirmed with western blot and RT-PCR after 6 days of culture prior to xenograft injection.

The pcDNA3.1 and pAPOC1 plasmids were obtained from Hanheng (Wuhan, China). All cell transfections were carried out in 6-well plates. Cells were transfected with each plasmid at 5ug per well by Lipofectamine 2000 (Invitrogen, Massachusetts, USA). miR-660-3p mimic and miR-660-3p mimic inhibitor were transfected with 80ng per well in the same way. After 48 h transfection, cells were collected for subsequent analysis.

### Western blot

Western blot analysis was performed as described previously.^50^ The primary antibodies used include APOC1 (Cat No. A5629, Abclonal, Wuhan, China), TGFβ2 (Cat No. A3640, Abclonal), SMAD3 (A19115, Abclonal), phospho-SMAD3 (Cat No. AP0727, Abclonal), Ki67 (Cat No. 27309–1-AP, Proteintech, Wuhan, China), and GAPDH (Cat No. 60004–1-Ig, Proteintech).

### Cell proliferation, migration, and invasion

Cell proliferation was assessed by the MTT assay as described previously.^50^ Cell invasion and migration were assessed using the transwell assay according to the manufacturer’s protocol (B.D. Bioscience). In brief, cells were pretreated with serum-free DMEM medium for 6 h and then were trypsinized and resuspended in serum-free DMEM medium. Cells of 5 × 10^4^ per well were plated onto the upper chamber without (migration) or with matrigel (invasion) and cultured for 15 h. The invaded cells on the submembrane surface were stained with crystal violet staining solution, and the number of cells in five randomly selected fields per well was counted.

### Quantitative real-time PCR

RNA extraction and subsequent steps had been described previously.^50^ The primers used are listed in Table S1.

### Dual-luciferase reporter assay

We constructed the wild-type APOC1 3’-UTR and mutant APOC1 3′-UTR using the pcDNA3.1 Dual-Luciferase Reporter Vectors (Promega). miRNA-660-3P mimic or miRNA-660-3p mimic inhibitor (Tsingke Biotechnology Co., Ltd. Wuhan, China) was transfected at 80 ng with an incubation period of 48 h using the Lipofectamine 2000 (Invitrogen).

### EdU cell proliferation assay

Cell proliferation was assessed by seeding cells in 24-well plates, incubated with 50 μM EdU for 2 h, and fixed in 4% paraformaldehyde and 0.5% Triton X-100 permeation according to the manufacturer’s protocol (EdU Cell Proliferation Kit with Alexa Fluor 488, Meilun, Guangzhou, China). Cells were visualized and imaged using a laser confocal microscope (Olympus, Japan) after counterstaining the nucleus with Hearst dye (Proteintech, Wuhan, China). The semi-quantitative (EdU positive cell percentage) results were detected by ImageJ.

### Immunofluorescence

Cells in 12-well plates were first fixed with 4% paraformaldehyde at 37°C for 30 min, permeated with 0.1 M PBS containing 0.2% Triton X-100 for 15 min, preincubated with 5% BSA for 30 min, and then incubated with anti-Ki67 primary antibody (1:200; Proteintech) at 4°C for 12 h. Next, cells were washed with PBS and incubated with the secondary antibody (1:500) for 1 h (CoraLite 488-conjugated Goat Anti-Rabbit IgG(H+L), Proteintech, Wuhan, China). Finally, cells were visualized and imaged using a laser confocal microscope (Olympus, Japan) after counterstaining the nucleus with Hearst dye (Proteintech, Wuhan, China). The Mean Fluorescence Intensity (MFI) results were detected by ImageJ.

### Immunohistochemistry

Immunohistochemistry was described in our previous study.^50^ Antibodies for anti-APOC1 (1:200) and anti-Ki67 (1:300) were purchased from Proteintech (Wuhan, China). The images were obtained by using a Leica fluorescence microscope (Germany).

### Tumor xenografts

The 4-week-old male nude mice were obtained from Shulaibao Biological Technology Co., Ltd (Wuhan, China). All experiments were conducted by the Chinese Animal Welfare Law and were approved by the Ethics Committee of the Second Affiliated Hospital of Wenzhou Medical University. The lv-shAPOC1-U87, lv-miR-660-3p-U87, lv-miR-660-3p-U251 and the corresponding control cells were injected subcutaneously into the middle lateral side of the axilla at the cell concentration of 3 × 10^6^/100 ul PBS. Tumors were measured externally with a caliper every 7 days before the final termination of mice on day 28 when tumors were excised and their weight and diameter were measured. Tumor volume (V) was calculated as follows: V = 0.5 × longitudinal diameter × latitudinal diameter. The largest and smallest tumors in each group were excluded for figure performance, as well as all tumors were included for statistical analysis.

### Statistical analysis

Each experiment was carried out in triplicate unless otherwise stated. Data are presented as means ± standard deviation (S.D.). Analyses comparing every two groups were performed with a Student’s t-test. Differences within the group were determined by one-way ANOVA. *P* < .05 was considered statistically significant.

## Supplementary Material

Supplemental Material

Supplemental Material

## Data Availability

The data used to support the findings of this study are available from the corresponding author upon request.

## Credit author statement

Jian Lin and Liang Yang: Conceptualization, Methodology, Software. Zhenkai Sun and Yuxi Rong: Data curation.Writing- Original draft. Jian lin and Zelin Yang, lnvestigation, Methodology. Chenglian Bai: Supervision.: Qiaoxiang Dong: Software, Validation. Jian Lin, Qiaoxiang Dong: Writing- Reviewingand Editing. All authors have read and agreed to the published version of the manuscript.
