# Supplementary material for: miRNA-660-3p inhibits malignancy in glioblastoma via negative regulation of APOC1-TGFβ2 signaling pathway
Source: Cancer Biol Ther. 2023 Nov 19;24(1):2281459. doi: 10.1080/15384047.2023.2281459 (PMC10783846; doi:10.1080/15384047.2023.2281459)
Supplement: Supplemental Material [file KCBT_A_2281459_SM5372.docx]

| Gene | Forward | Reverse |
| --- | --- | --- |
| miR-660-3p  specific primer | 5’-CGACCTCCTGTGTGCATGGATTA-3’ |  |
| SMAD7 | 5’-TTCCTCCGCTGAAACAGGG-3’ | 5’-CCTCCCAGTATGCCACCAC-3’ |
| HMAG2 | 5’-CAGCAGCAAGAACCAACCG-3’ | 5’-TGTTGTGGCCATTTCCTAGGT-3’ |
| TGFβ2 | 5’-CGACGAAGAGTACTACGCCA-3’ | 5’-GATGGCATTTTCGGAGGGGA-3’ |
| LRP6 | 5’-ACGATTGTAGTTGGAGGCTTG-3’ | 5’-ATGGCTTCTTCGCTGACATCA-3’ |
| SMG6 | 5’-ACCAAGACGACATCAAGGTGT -3’ | 5’-AGCATCCAATCTGACCAGACTT-3’ |
| SIRT7 | 5’-ACGCCAAATACTTGGTCGTCT-3’ | 5’-AGCACTAACGCTTCTCCCTTT-3’ |
| APOC1 | 5’-AGGACAAGGCTCGGGAACTCAT-3’ | 5’-GATGTCACCCTTCAGGTCCTCA-3’ |
| GAPDH | 5’-AGGACAAGGCTCGGGAACTCAT-3’ | 5’-GATGTCACCCTTCAGGTCCTCA-3’ |

**Table S1: Primers used in PCR**

**Table S2: Sequence of miR-660-3p mimic and miR-660-3p inhibitor**

| miR-660-3p mimic | 5′-ACCUCCUGUGUGCAUGGAUUA-3′ |
| --- | --- |
| Mimic negative control | 5′-UUCUCCGAACGUGUCACGUTT-3′ |
| miR-660-3p inhibitor | 5′-UAAUCCAUGCACACAGGAGGU-3′ |
| Inhibitor negative control | 5′-CAGUACUUUUGUGUAGUACAA-3′ |
