# Supplementary figures and images for: miRNA-660-3p inhibits malignancy in glioblastoma via negative regulation of APOC1-TGFβ2 signaling pathway
Source: Cancer Biol Ther. 2023 Nov 19;24(1):2281459. doi: 10.1080/15384047.2023.2281459 (PMC10783846; doi:10.1080/15384047.2023.2281459)

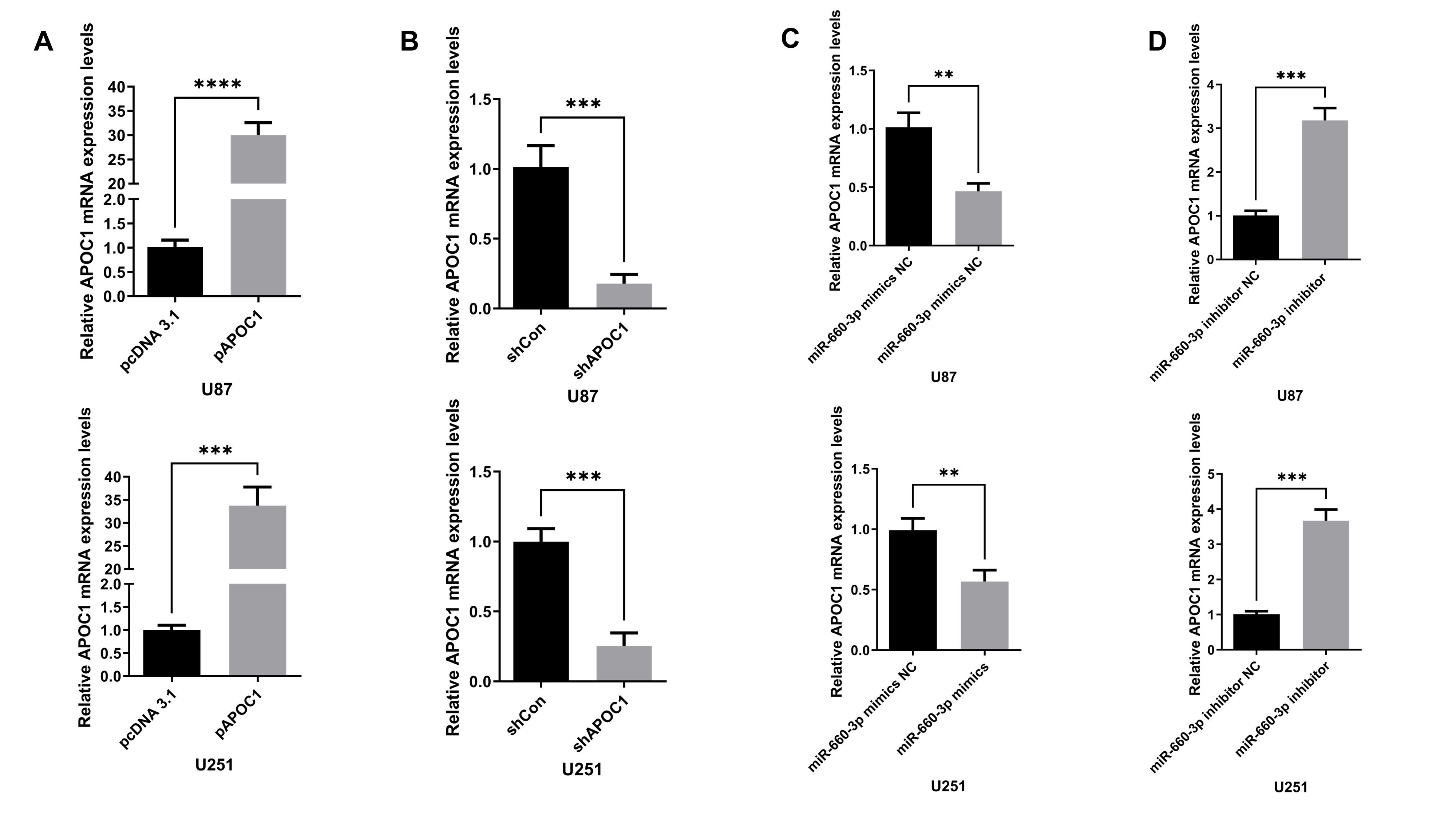

Supplement: Supplemental Material [file KCBT_A_2281459_SM2659.zip › Supplemental figures/FIG S1.png]

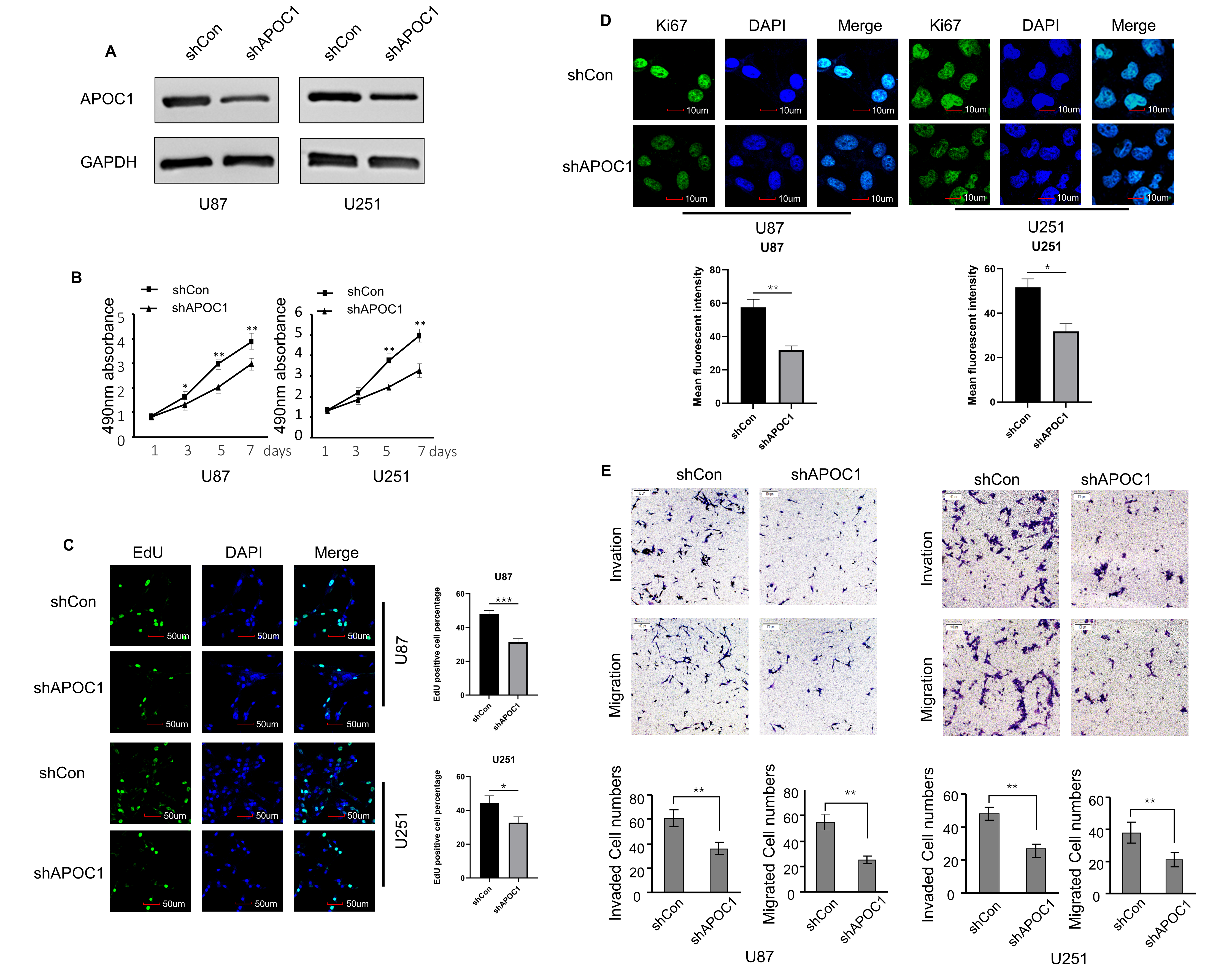

Supplement: Supplemental Material [file KCBT_A_2281459_SM2659.zip › Supplemental figures/FIG S2.png]

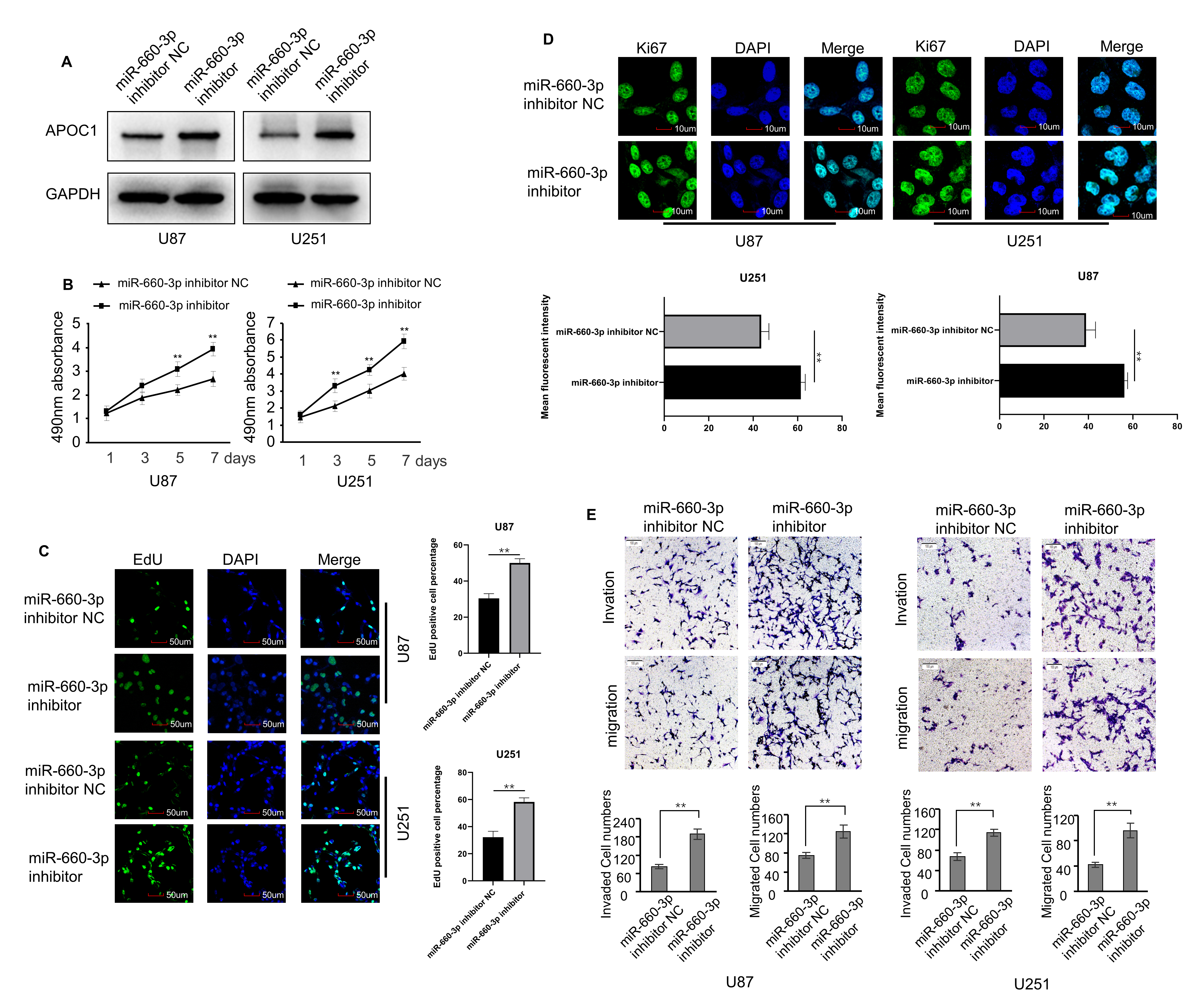

Supplement: Supplemental Material [file KCBT_A_2281459_SM2659.zip › Supplemental figures/FIG S3.png]

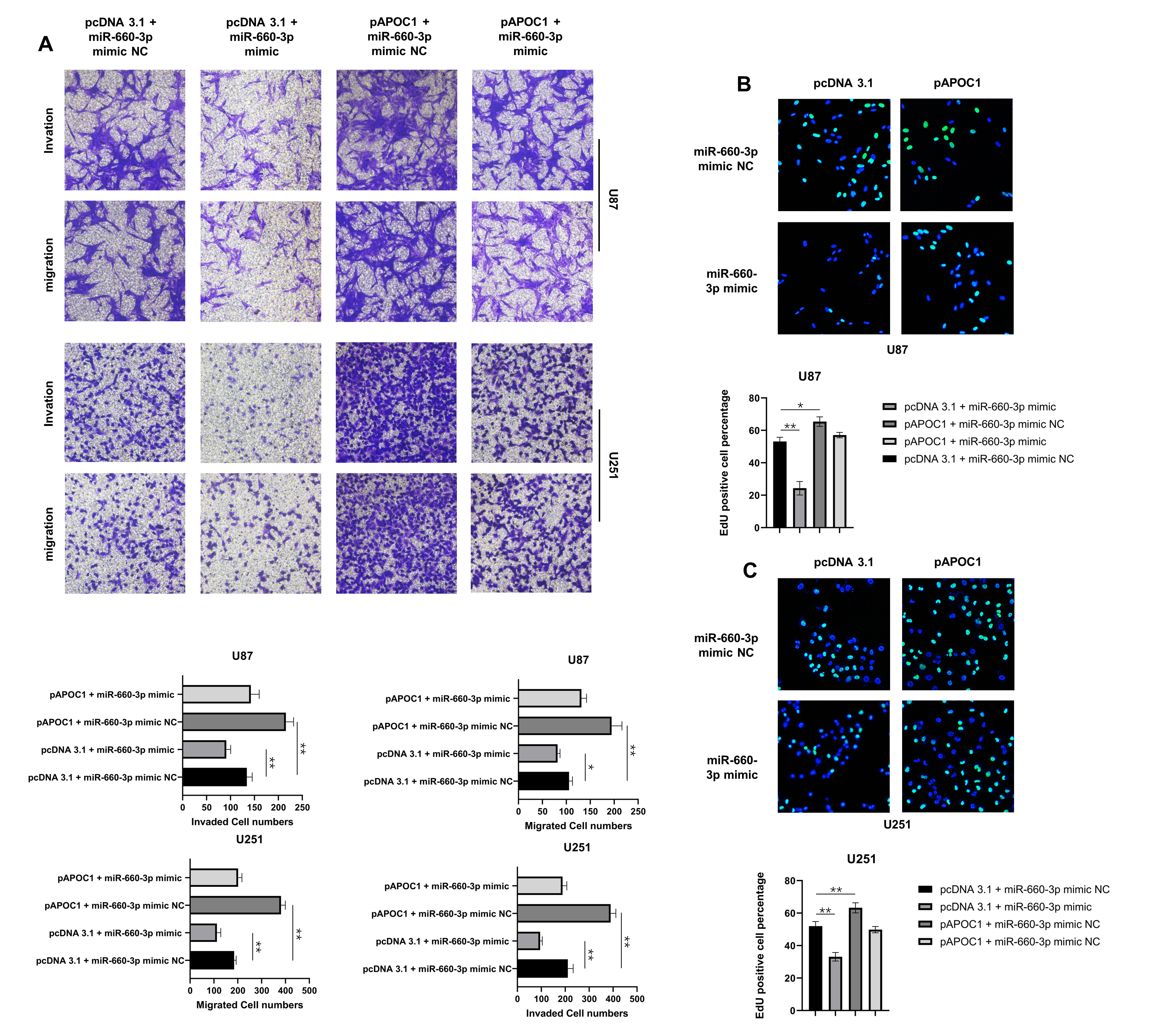

Supplement: Supplemental Material [file KCBT_A_2281459_SM2659.zip › Supplemental figures/FIG S4.png]
